# Alterations in Emotional Diversity Correspond With Increased Severity of Attenuated Positive and Negative Symptoms in the Clinical High-Risk Syndrome

**DOI:** 10.3389/fpsyt.2021.755027

**Published:** 2021-12-23

**Authors:** Zachary Anderson, Tina Gupta, William Revelle, Claudia M. Haase, Vijay A. Mittal

**Affiliations:** ^1^Department of Psychology, Northwestern University, Evanston, IL, United States; ^2^School of Education and Social Policy, Northwestern University, Evanston, IL, United States

**Keywords:** psychosis, clinical high-risk, attenuated positive symptom syndrome, emotion, emotional diversity

## Abstract

**Background:** Alterations in emotional functioning are a key feature of psychosis and are present in individuals with a clinical high-risk (CHR) syndrome. However, little is known about alterations in emotional diversity (i.e., the variety and relative abundance of emotions that humans experience) and clinical correlates in this population.

**Methods:** Individuals meeting criteria for a CHR syndrome (*N* = 47) and matched healthy controls (HC) (*N* = 58) completed the modified Differential Emotions Scale (used to derive scores of total, positive, and negative emotional diversity) and clinical interviews (i.e., Structured Interview for Psychosis-Risk Syndromes).

**Results:** Findings showed that the CHR group experienced lower levels of positive emotional diversity compared to HCs. Among the CHR individuals, lower levels of positive and higher levels of negative emotional diversity were associated with more severe attenuated positive and negative symptoms. Analyses controlled for mean levels of emotion and current antipsychotic medication use.

**Discussion:** Results demonstrate that altered emotional diversity (in particular lower levels of positive and higher levels of negative emotional diversity) is a clinically relevant marker in CHR individuals, above and beyond alterations in mean levels of emotional experiences. Future studies may probe sources, downstream consequences, and potential modifiability of decreased emotional diversity in individuals at CHR.

## Introduction

Emotional dysfunction is a key feature of psychotic disorders such as schizophrenia (1, 2), which often manifests in mood symptomatology (3) and can be characterized by negative symptoms (4–7). Recent work explores how emotion-related problems may influence the onset of psychotic disorders by studying emotive dysfunction in those who meet criteria for a clinical high-risk (CHR) syndrome. This is a rich area of investigation as a subgroup of CHR individuals may develop a first-episode of psychosis in a short window of time (8, 9). Despite mounting interest in studying altered emotional functioning in those with a CHR syndrome (10–12), existing studies in CHR groups are primarily focused on documenting differences in mean levels of positive and negative emotion (13) and do not account for metrics that reflect other underlying emotion-related processes. One potential avenue that can inform this gap is through examining aspects such as emotional diversity [i.e., the variety and relative abundance of emotions that humans experience (14)], which is reliably altered in clinical populations (15) and may illuminate altered emotion-related processes in those with a CHR syndrome. Additionally, specific patterns of discrete emotions may be more sensitive to differences that predispose individuals to particular clinical experiences. Such work stands to further scientific understanding of the conceptualization of emotional processes, prevention strategies, and targeted emotion-based treatments in this group.

Emotion-related symptoms often co-occur with positive psychotic symptoms (3) as well as negative symptoms that characterize many patients with schizophrenia (4–7, 16). While work investigating emotional diversity in groups diagnosed with schizophrenia is limited, there is evidence of reductions in the ability to differentiate various emotional states (e.g., positive from negative emotional states) (11) and alterations in positive and negative emotional experiences (17). However, this is not the whole picture. Scientists have long noted that not all positive and negative emotions are created equal (18). Pride, for example, is a positive emotion that is associated with postural expansion and achievement, while joy is more closely tied to the pursuit of reward (19, 20). Anger is a negative emotion that is a response to an offense against me or mine, whereas sadness might follow an irrevocable loss (21). Thus, different positive and negative emotion differ in what stimuli elicits them, what they feel like, and what their consequences are (22, 23). This granularity in emotional experience may have utility in characterizing various psychotic disorders and, in particular, may have value for understanding subtle emotional changes that may be associated with risk for the future development of a psychotic disorder.

Similar to work in schizophrenia, studies focusing on composites of positive and negative emotion have led to foundational findings related to the CHR syndrome (10, 11, 13, 17). A series of studies in CHR populations repeatedly tie deficits in emotional experience to risk for psychosis, other central symptoms, and declines in functioning (11, 24–27). However, recent work that relates emotional diversity to a variety of clinical symptoms suggests that commonly used composites may not fully describe emotions' role in developing psychopathology (15, 28). This idea, based on early work on the Shannon entropy metric (29), led to a measure that captures the variety and relative abundance of self-reported emotions that humans experience—emotional diversity (14). Person A, for example, may have low positive emotional diversity in that she experiences very high levels of pride, but few other positive emotions. Person B may have high levels of positive emotional diversity in that she experiences an abundance of different positive emotions, such as gratitude, interest, joy, love, as well as pride. Research in several samples has shown that higher levels of emotional diversity predict a variety of positive outcomes including reduced depression severity, fewer hospital visits, improved physical health, and better outcomes following daily challenges or unresolved personal conflicts (15, 30, 31). Such research posits that a diverse emotional experience reflects the existence of intact cognitive mechanisms that may serve a variety of roles that promote healthy living. However, the extent to which emotional diversity may be altered in CHR groups is unknown.

Why look at the complexity of different emotional experiences as opposed to traditional averages of positive and negative emotion? The answer to this question revolves, primarily, around the issue of variability in the experience of specific emotions. Many situations we encounter in everyday life do not elicit just only one emotion but are complex and ambiguous in nature and thus may give rise to multiple emotions. Consider someone who cooks a delicious dinner for a friend. Person A may experience primarily pride in this situation and may want to celebrate their accomplishment. Person B, however, may experience not only pride but also gratitude (because the friend brought a wonderful dessert), interest (because they are curious to learn what their friend has been up to), joy (because of the festive occasion), and love [because of the shared positive connection (32)] and, thus, may also want to thank the friend, ask them how they are really doing, smile, and experience closeness. This heterogeneity in an individual's emotional experiences highlights the utility of access to a diverse set of emotions and illustrates the impact that subtle differences in internal emotions can have on behavior and downstream functioning (33, 34).

Increased use of emotional diversity and related constructs can be traced to past work that seeks to understand the role of emotion in creating complex mental representations of these everyday occurrences (14, 34, 35). This work underscores the benefits of access to diverse emotions, which act as building blocks used to characterize complex life events and generate a conscious experience that is flexible to a variety of possible sensory inputs (33). This process is thought to be a function of an individual's ability to identify particular emotions or emotional awareness (36, 37) and the richness or emotional complexity of an individual's reported experience (28, 38). When these two components are present, it is argued that individuals may be better able to interact with the surrounding world. Said another way, rich and diverse emotional experiences allow for the construction of a more complex “mentalization” network, or an internal representation of real-life situations that individual's confront in everyday life (33, 34). These situational constructions are hypothesized to be central to one's ability to regulate behavior during difficult situations as they allow for more precise mapping of the emotional components of a current situation to past occurrences (34). Emotional diversity can be thought of as a composite value that describes the components necessary for these complex mentalizations and current work documents mental and physical health benefits in individuals who exhibit greater emotional diversity (14, 15). If applied to psychosis spectrum disorders, these metrics could yield important information related to dysfunctional cognitive mechanisms at the core of psychotic symptoms. In particular, the degree of granularity offered by metrics of emotional diversity could be helpful in identifying the kinds of subtle changes in emotional experience that partially characterizes the CHR syndrome.

In addition to studying the diversity of an individual's emotional experience, it is equally important to understand how individual emotions might relate to specific presentations of central symptoms. Recent advances in the personality literature provide a method for exploring this issue using the predictive power of individual items (e.g., “I get overwhelmed by emotions”) in place of large composites such as the big 5 personality traits (39). This work draws inspiration from genetics research where individual genes are shown to contribute small pieces of information that, together, accurately predict certain phenotypes. This forms a conceptual bridge to recent work that explores differences in specific emotion items and their relationship with attenuated symptoms in CHR groups (13). However, this new algorithm may provide more sensitivity as it is designed to extract patterns of experience that optimally predict a desired criterion. Such an approach could complement studies that document reductions in emotional diversity as it may identify particular emotions that become the epicenter of an individual's experience. It may also shed light on some inconsistencies in the reports of emotion-related changes underlying psychosis (12).

The present study examined emotional diversity and discrete emotion items in a sample of individuals meeting criteria for a CHR syndrome and healthy controls (HC) along with their clinical correlates. Despite mounting interest in altered emotional functioning related to symptoms of the CHR syndrome (10–12), the emphasis of past work on mean levels of positive and negative emotional experiences (13) leaves a question of whether changes in emotional diversity might characterize CHR groups. Recent work that defines subgroups on the basis of individual symptoms (27, 40, 41) also highlights the importance of incorporating item level emotion profiles that may provide more specific targets for intervention and future research.

The current study tested the following hypotheses. First, based on findings of reductions in the ability to differentiate positive and negative emotional states in schizophrenia patients (42) and reduced flexibility and diversity in other domains of functioning in psychosis and CHR more broadly (43), group comparisons were used to test whether the CHR group would show lower emotional diversity (positive, negative, and total) compared to healthy controls. In addition, based on findings that document the benefits of positive emotional diversity in psychopathology (15, 30), a general linear model was used to test whether positive emotional diversity related to less severe positive and negative symptoms in CHR individuals. Links between negative emotional diversity and clinical symptoms were also examined in those with a CHR syndrome. We were reluctant to formulate a hypothesis in light of mixed findings of the adaptiveness of negative emotional diversity in healthy individuals (14) vs. individuals with psychopathology (15). Models related to emotional diversity included a control variable describing mean levels of positive and negative emotion, respectively. Finally, based on recent work documenting the predictive efficacy of individual items over composites (39), a statistical learning approach was used to test whether specific attenuated symptoms in this sample corresponded with unique profiles of discrete emotions.

## Methods

### Participants

Forty-seven participants meeting criteria for a CHR syndrome and 58 age matched healthy controls were recruited who were between the ages of 13 and 21 (*M* = 19.15, *SD* = 2.20). All participants were recruited through the Adolescent Development and Preventive Treatment (ADAPT) research program through internet, newspaper, public transportation advertisements, email postings, and community professional referrals. Exclusion criteria included the presence of head injury or a neurological disorder, lifetime substance dependence, IQ < 70, and/or a lifetime diagnosis of a schizophrenia spectrum disorder or mood disorder with psychosis. Three additional participants were excluded who either (1) converted to psychosis shortly after participating in the study or (2) developed attenuated symptoms after participating as a HC subject. In our sample, there were seven individuals taking antipsychotic medications. To improve the generalizability of findings, these individuals were included in our final analyses, in line with previous studies in this area (13). Current antipsychotic use was included in all analyses as a covariate in the present work to control for potential effects.

### Clinical Interviews

Participants were given the Structured Interview for Psychosis-Risk Syndromes (SIPS) (44) to determine CHR criteria. This scale assesses severity in addition to the frequency, duration, distress, impact, and conviction of symptoms, weighing each of these factors into a single score for each of five positive symptoms domains including P1 (unusual thought content/delusional ideas), P2 (suspiciousness/persecutory Ideas), P3 (grandiose ideas), P4 (perceptual abnormalities/hallucinations), and P5 (disorganized communication). This scale also assessed severity of six negative symptoms including N1 (social anhedonia), N2 (avolition), N3 (expression of emotion), N4 (experience of emotions and self), N5 (ideational richness), and N6 (occupational functioning). Criteria for CHR status included receiving a rating of a 3 (moderate) to 5 (moderately-severe) on at least one positive symptom domain (e.g., unusual thought content/delusional ideas). This score was determined as a function of the severity of each symptom, which considers social function, occupational function, and clinical distress, as well as the frequency of each symptom (i.e., symptoms must be present at least once per week for at least the past month). Attenuated positive and negative symptom scores reflect both item-level and sum scores. Participants also qualified for the CHR group if they met diagnostic criteria for schizotypal personality disorder and/or had a family history of psychosis (e.g., first-degree relative with a psychotic disorder such as schizophrenia) with a decline in functioning. These scores acted as the primary measure of the severity of attenuated psychotic symptoms in the current study. Global functioning was assessed with the Global Functioning Scale in the SIPS, which provides metrics for both social (e.g., number of friends, how often the individual engages in social activity) and role functioning (e.g., performance at work/school). Additionally, the Structured Clinical Interview for the DSM-IV (45) was given to rule out the presence of a psychotic disorder and further assess Schizotypal Personality Disorder, if needed.

### Emotional Diversity

The modified Differential Emotions Scale (mDES) (46) was used as the primary measure of trait-like experiences of various emotions. Participants rated the degree to which they felt each emotion on average or in general. Ratings were made on a 5 point Likert-like scale that ranged from 0 (not at all) to 4 (extremely). This modified version of the original Differential Emotion Scale was used as it increases the number of positive emotions from 3 to 10. These items include amusement, awe, content, joy, gratitude, hope, interest, love, pride, and surprise. Negative affect items include anger, shame, fear, disgust, embarrassment, guilt, sadness, and contempt.

Once these discrete emotions were measured, emotional diversity was calculated in the following way:

1. To better reflect the full range of responses, rescaled ratings to range from −2 to 2 and then performed a logistic transformation.
2. Divided the intensity of an individual emotion by the sum of intensities across all types of emotions.
3. Multiplied this proportion by its natural log.
4. Repeated for each specific emotion.
5. Summed values across all emotions to calculate total emotional diversity.
  a. Summed values across all positive emotions to calculate positive emotional diversity.
  b. Summed values across all negative emotions to calculate negative emotional diversity.

This provides a composite value that describes the relative abundance of total, positive, and negative emotions. For an example calculation of emotional diversity with simulated data see Supplementary Material.

### Data Analysis Plan

All analyses were performed in R using the *psych* package (47, 48). Chi-squares/One-way ANOVAs were used as appropriate to test for demographic differences between groups. The primary aim of this project was to identify group level differences in emotional diversity measures, which were calculated using methods described by Quoidbach et al. (14) and correspond with total, positive, and negative emotional diversity. A general linear model (GLM) approach was used to test for group differences while controlling for current antipsychotic medication as well as respective levels of mean positive and negative emotion. Next, a GLM was used to relate composites of emotional diversity to total attenuated positive and negative symptom severity. As in the previous model, this analysis controlled for current antipsychotic medications and respective levels of mean positive and negative emotions. Our final exploratory aim was to determine whether unique profiles of discrete emotions related to specific positive and negative symptoms. A previously developed algorithm (49) was used to find a parsimonious set of discrete emotions on the basis of cross-validated, unit-weighted zero-order correlations that optimally account for variance across all attenuated positive and negative symptoms. This analysis included the following symptoms from the SIPS interview: P1 (unusual thought content/delusional ideas), P2 (suspiciousness/persecutory Ideas), P3 (grandiose ideas), P4 (perceptual abnormalities/hallucinations), P5 (disorganized communication), N1 (social anhedonia), N2 (avolition), N3 (expression of emotion), N4 (experience of emotions and self), N5 (ideational richness), and N6 (occupational functioning).

## Results

### Demographics

Descriptive statistics for demographic variables are presented in Table 1. There were no significant group differences in age, biological sex, or parent education. Nine of the CHR participants (19%) were taking antipsychotic medication at the time of the study. As expected, the CHR group showed elevated ratings on total attenuated positive symptoms and total negative symptoms (Table 1).

**Table 1:** Demographic details of a sample of individuals with a clinical high-risk syndrome and controls.

	**CHR**	**Control**	**Statistic**	***p***
Age	19.04 (1.63)	19.24 (2.58)	*t* = −0.481	0.63
**Biological sex**				
Male	26	22	χ^2^ = 2.50	0.11
Female	21	36		
Total	47	58		
Parent education	15.66 (2.20)	15.71 (2.68)	*t* = −0.09	0.93
Hispanic	12	12		
**Race**				
First nations	2	0		
East Asian	2	5		
Southeast Asian	0	2		
South Asian	0	0		
Black	0	2		
Central/South American	8	11		
West/Central Asia and Middle East	1	0		
White	31	37		
Native Hawaiian or Pacific Islander	0	0		
Interracial	3	1		
**Symptoms**				
Total positive	11.45 (4.55)	0.41 (1.03)	*t =* 16.30	< 0.001
Total negative	9.28 (7.15)	0.24 (0.54)	*t =* 8.64	< 0.001

*CHR, Clinical high-risk; Means and standard deviations are presented for age, parental education, and symptoms; Age and parental education are presented in years. Clinical symptoms correspond with summed scores from the Structured Interview for Psychosis-Risk Syndromes (SIPS) and reflect overall severity of attenuated positive and negative symptoms*.

### Group Differences in Emotional Diversity

**Figure 1:**
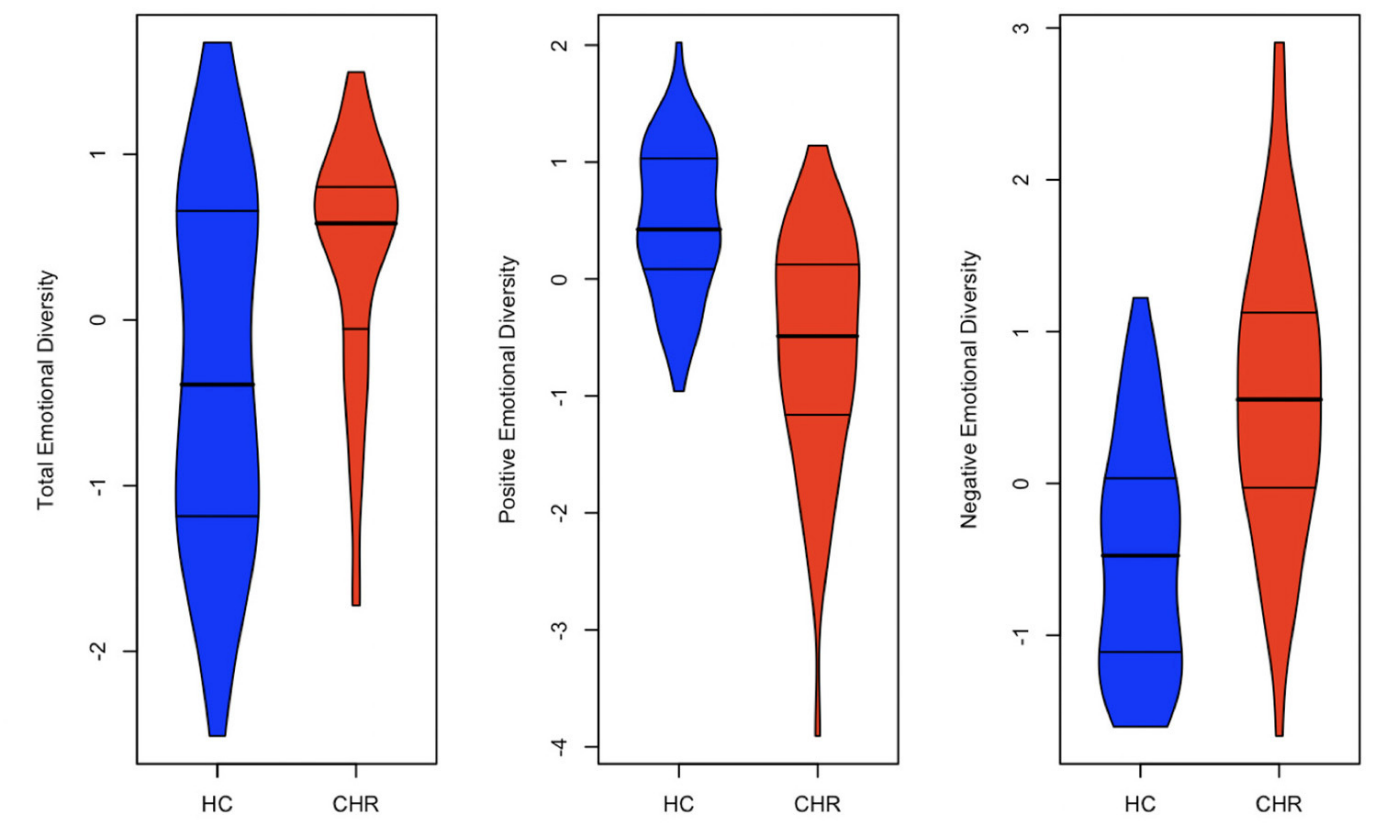
Group differences in emotional diversity. The violin plots above visualize group differences in three standardized unadjusted emotional diversity metrics (total, positive, and negative). Results indicate an effect for positive emotional diversity but not total or negative emotional diversity.

Findings revealed significantly lower positive emotional diversity in the CHR group compared with healthy controls (*b* = −0.35, 95% C.I. [−0.48, −0.21], *R*^2^ = 0.198, *p* < 0.001) (Figure 1). Findings also revealed heightened negative emotional diversity in the CHR group compared with the healthy controls (*b* = 0.08, 95% C.I. [0.00, 0.15], *R*^2^ = 0.037, *p* = 0.050) (Figure 1). Lastly, with respect to total emotional diversity, there was no difference between CHR group and the healthy controls (*b* = 0.00, 95% CI [−0.17, 0.18], *R*^2^ <0.001, *p* = 0.960) (Figure 1).

### Emotional Diversity and Clinical Symptoms

When exploring the relationships between total positive and negative symptom severity and their scores on total, positive, and negative emotional diversity measures, findings reveal no relationship between positive symptom severity and total emotional diversity (*b* = −0.09, 95% C.I. [−0.32, 0.14], *R*^2^ = 0.006, *p* = 0.420) and a significant negative relationship between negative symptom severity and total emotional diversity (*b* = −0.32, 95% C.I. [−0.51, −0.13], *R*^2^ = 0.100, *p* = 0.001). Next, we looked at the specific relationship between positive emotional diversity and symptom severity above and beyond the effect of mean levels of positive emotion. Findings indicated a negative relationship between positive symptom severity and positive emotional diversity (*b* = −0.66, 95% C.I. [−0.87, −0.45], *R*^2^ = 0.277, *p* < 0.001) and a negative relationship between negative symptom severity and positive emotional diversity (*b* = −0.69, 95% C.I. [−0.87, −0.50], *R*^2^ = 0.343, *p* < 0.001). Finally, with respect to negative emotional diversity, results suggest a significant positive relationship between negative emotional diversity and positive symptom severity (*b* = 0.59, 95% C.I. [0.12, 1.06], *R*^2^ = 0.057, *p* = 0.015) as well as a significant positive relationship with negative symptom severity (*b* = 0.49, 95% C.I. [0.08, 0.90], *R*^2^ = 0.052, *p* = 0.020).

### Prediction of Positive and Negative Symptoms From Emotion Items and Composites

These results reflect the final exploratory aim of this paper that seeks to identify unique patterns of discrete emotions that maximally predict attenuated positive and negative symptom severity. Across the majority of symptoms, some items appear common (e.g., “I feel angry, irritated, annoyed” “I feel sad, downhearted, unhappy”). Each discrete positive emotion is negatively correlated with attenuated positive and negative symptom severity, while negative emotions are negatively correlated with each of the attenuated positive and negative symptoms. There are some attenuated positive symptoms (e.g., suspiciousness/persecutory ideas) that are not accounted for well by this method, but others appear have relatively strong correlations with several discrete emotions. To illustrate this point, Table 2 presents the top three emotions that correlate most highly with each symptom. Supplementary Table 2 documents all emotion items that were correlated with each attenuated positive and negative symptom across the 10 fold cross validation procedure. Critically, these values do not reflect the independent effect of each emotion as is typical of beta weights but rather reflects the highest item level zero order correlations. As can be seen in Table 2, several clinical symptoms share a most highly correlated item, such as grandiose ideas and perceptual abnormalities/hallucinations where feelings of gladness, anger, and sadness appear in both symptoms' most highly correlated emotions. However, distinction between each symptom appear in emotion items, which are less strongly correlated with each symptom (Supplementary Table 2). Some examples of these include grateful (*r* = −0.34) and ashamed (*r* = 0.32) for Grandiose Ideas as well as disgust (*r* = 0.42) and hope (*r* = −0.41) for Perceptual Abnormalities/Hallucinations. Similar distinctions exist across each of the attenuated positive and negative emotion categories.

**Table 2:** Top correlations between discrete emotion items across all attenuated positive and negative psychotic symptoms.

**Attenuated positive/negative symptom**	**Emotion item 1**	**Emotion item 2**	**Emotion item 3**
Unusual thought content/delusional ideas	Sad *r =* 0.52 (0.03)	Angry *r =* 0.51 (0.02)	Glad *r* = −0.48 (0.03)
Suspiciousness/persecutory ideas	Guilty *r =* 0.29 (0.05)	Scared *r* = 0.26 (0.03)	Embarrassed *r* = 0.24 (0.05)
Grandiose ideas	Glad *r* = −0.41 (0.03)	Angry *r* = 0.39 (0.03)	Sad *r* = 0.37 (0.04)
Perceptual abnormalities/hallucinations	Glad *r* = −0.48 (0.03)	Angry *r* = 0.47 (0.02)	Sad *r* = 0.46 (0.03)
Disorganized communication	Angry *r* = 0.43 (0.02)	Scared *r* = 0.40 (0.03)	Sad *r* = 0.38 (0.02)
Social anhedonia	Sad *r* = 0.56 (0.03)	Angry *r* = 0.48 (0.02)	Contempt *r* = 0.45 (0.03)
Avolition	Sad *r* = 0.61 (0.02)	Angry *r* = 0.57 (0.01)	Glad *r* = −0.56 (0.02)
Expression of emotion	Sad *r* = 0.52 (0.02)	Angry *r* = 0.48 (0.02)	Glad *r* = −0.47 (0.02)
Experience of emotions and self	Disgust *r* = 0.59 (0.01)	Angry *r* = 0.58 (0.02)	Glad *r* = −0.58 (0.02)
Ideational richness	Sad *r* = 0.43 (0.03)	Angry *r* =0.41 (0.02)	Ashamed *r* = 0.39 (0.02)
Occupational functioning	Sad *r* = 0.56 (0.02)	Glad *r* = −0.52 (0.02)	Angry *r* = 0.50 (0.02)

*Each correlation represents the mean zero-order correlation generated across a 10-fold cross validation procedure. Standard deviations of these estimates can be found in parentheses. The three most highly correlated items for each symptom are presented along with average zero order correlation coefficients and their corresponding standard deviations of these estimates across this cross validated analysis*.

## Discussion

The current work explored several constructs underlying emotional experience in a sample of individuals with a CHR syndrome. To this end, we first explored differences in total emotional diversity, emotional diversity specific to positive emotions, and emotional diversity specific to negative emotions. The CHR group was primarily associated with lower levels of positive emotional diversity and higher levels of negative emotional diversity. Dimensional measures of attenuated positive and negative symptoms had strong negative relationships with positive emotional diversity and strong positive relationships with negative emotional diversity. Finally, we report profiles of emotions that correspond with specific positive (e.g., unusual thought content/delusional ideas) and negative (e.g., social anhedonia) symptoms.

The current work documents group changes in emotional diversity when comparing CHR and HC groups. These findings include a significantly reduced positive emotional diversity and marginally elevated negative emotional diversity. This adds to a literature that has often documented differences in mean emotional experience (10, 13), by demonstrating that, above and beyond changes in mean emotion, the relative abundance of particular emotions also appears to characterize the CHR syndrome. Emotional diversity accounts for a significant amount of variance related to clinical correlates of the CHR group. This effect is characterized by a negative relationship between positive emotional diversity and attenuated positive and negative symptom severity as well as a positive relationship between negative emotional diversity and attenuated positive and negative symptoms. This finding maps onto a growing literature that documents the protective nature of diverse positive emotions (14, 15) and may reflect the existence of intact emotion and cognitive mechanisms that protect against the development of psychopathology (15).

In this population, along with changes in positive symptoms, clinical progression has been characterized by worsening negative symptoms, including anhedonia (50). However, patients with schizophrenia and other illnesses along the psychosis spectrum have reported similar levels of positive emotion in response to pleasant stimuli and show normative responses to rewarding outcomes (12). This creates a paradox where hedonic reward systems appear to be intact despite disruption in the function of other emotion related processes (12). Current results add to this paradox as increases in negative emotional diversity, or the relative abundance of negative emotions, corresponds with increased severity of negative symptoms that typically suggest broad reductions in emotional experience. On the other hand, the negative relationship between positive emotional diversity and attenuated positive and negative symptom severity maps more closely to what we might expect and is more in line with current work that describes a reduction in positive emotions (12). Taken together, the current findings could suggest that progression toward psychotic illness can be characterized as a kind of funnel where negative emotional experiences begin to play a stronger role in an individual's emotional experience, while protective positive emotions become less abundant.

To complement findings related to emotional diversity, we generated profiles of discrete emotions to identify emotions that may share unique relationships with particular attenuated positive and negative psychotic symptoms. To our knowledge, the current study is the first to evaluate relationships between profiles of discrete emotions and attenuated positive and negative symptoms in the CHR syndrome using statistical learning methods. Some of these profiles map closely to what might be expected. Results from this work could suggest that particular symptoms are composed by a somewhat similar recipe of discrete emotions with critical distinctions that may confer increased risk for specific symptom. For example, feelings of guilt and fear appear relatively unique to suspiciousness/persecutory ideas, while other emotions (e.g., “I feel angry, irritated, annoyed” “I feel sad, downhearted, unhappy”) appear more common. This commonality aligns with previous investigations that have noted similar relationships between negative emotions and the CHR syndrome (13) as well as schizophrenia (17). The distinctions could suggest that attenuated positive and negative symptoms are constructed, in part, as a function of unique combinations of discrete emotions. The current study's design and sample size do not allow for a strong interpretation of these distinctions and future work may want to employ methods such as ecological momentary assessment to more accurately model fluctuations in emotional experience within this population. Despite these limitations, the current findings could imply that attenuated positive and negative symptoms are formed partly as a function of unique combinations of discrete emotions that construct the particular experiences that are associated with each respective symptom.

Emotions are considered core building blocks of conscious experience and have been theorized to allow for more accurate mental models of complex situations (33, 34). These more accurate representations are believed to aid in emotion regulation and have been correlated with improved mental and physical health outcomes (14, 15, 33, 35). The current results suggest a two part shift in emotions occurs during the progression of psychotic symptoms. The decrease in positive emotional diversity and the increase in negative emotional diversity could suggest that, as part of the early clinical progression, an individual's emotional landscape begins to shift from a balanced and flexible set of emotions to a more negatively valenced and conceptually restricted set of emotional experiences. Results demonstrating a similar set of emotions correlate with the majority of attenuated positive and negative symptoms could suggest that a similar emotional phenotype lies at the core of this shift. However, distinctions across the entire emotion profile of each symptom category hints at a more complex mechanism where particular emotions may be more strongly tied to different manifestations of the CHR syndrome. These discrete emotions may be helpful targets for intervention focused on CHR populations and future research might explore the relationship between specific emotions that might describe negative cognitions that reinforce negative beliefs about oneself and others (51, 52).

Emotional dysfunction in individuals with a CHR syndrome is a critical topic as research continues to identify emotion related mechanisms that characterize the progression of psychosis (12, 50, 53). The current work provides nuanced pieces of information in an effort to address the puzzle of emotional dysfunction in these groups. Extending previous work on emotion in clinical high-risk groups (10, 11, 13, 17), the current research highlights the importance of positive and negative emotional diversity and suggests that the experience of a diverse set of positive emotions may be protective against symptom development. These results can inform vulnerability models by integrating components of emotional diversity. Furthermore, these data support past work that suggests that higher levels of emotional diversity may enhance quality of life (14, 15). While the mechanisms underlying these findings are not fully understood, there are some possibilities. For example, drawing on cognitive behavioral theories (52, 54, 55), it is possible that cognitive mechanisms play a role in the identification and experience of a wide range of positive emotions (15, 30). Findings related to the statistical learning approach suggest that specific attenuated positive and negative symptoms in CHR groups may be defined by unique patterns of discrete emotional experiences. While speculative, progression toward psychosis may lead to a funneling of emotional experience where the energy that previously drove diverse positive and negative emotions becomes increasingly focused on particular negative emotions and give rise to particular sets of clinical symptoms (28). This is in line with some recent work that links negatively valenced cognitions to the progression of psychotic illness (52). The current findings also lay a foundation for future work to explore metrics related to emotional diversity and serve as initial evidence that mechanisms underlying diverse emotional experiences play a role in the progression of psychotic illness.

The present study has limitations that open the door for future research. First, while the approaches used in this work were effective at characterizing the current sample, the study used a cross-sectional design. Additional research is needed to assess findings over time, using longitudinal studies. Future work with prospective designs and larger sample sizes will also provide the needed power to elucidate the impact of antipsychotic medications in CHR individuals on emotion related metrics and their relationship with the longitudinal progression of psychosis. Next, although the mDES provides information about a wide range of emotions, it does not provide information about the subjective intensity of emotional experiences or how each emotion might vary throughout the day. Future work, particularly studies employing ecological momentary assessment, might consider adding a similar measure of discrete emotional experiences. With this, it is possible that information about the temporal dynamics of specific emotions may illuminate underlying cognitive processes that drive the changes in emotional experience noted in the current study. Additionally, while the current results imply dysfunction in underlying emotion-related mechanisms the current results cannot be used to make causal inferences. Future work might consider leveraging these findings in an effort to apply methods geared toward identifying the mechanisms that drive the current findings.

## Data Availability Statement

The raw data supporting the conclusions of this article will be made available by the authors, without undue reservation.

## Ethics Statement

The studies involving human participants were reviewed and approved by Human Research and Institutional Review Board at the University of Colorado, Boulder. Written informed consent to participate in this study was provided by the participants' legal guardian/next of kin.

## Author Contributions

ZA, TG, WR, CH, and VM contributed substantially to the conceptualization of the current work. ZA and WR ran analyses and prepared the final results. All authors were involved in drafting and revising various components of the manuscript, provided insight with respect to analyses present in the current work, approved the submitted version of this manuscript, and agreed to be accountable for all aspects of their work.

## Funding

The authors are grateful to have received support from the National Institutes of Health, which awarded Grant Number R21MH115231 to CH and VM.

## Conflict of Interest

The authors declare that the research was conducted in the absence of any commercial or financial relationships that could be construed as a potential conflict of interest.

## Publisher's Note

All claims expressed in this article are solely those of the authors and do not necessarily represent those of their affiliated organizations, or those of the publisher, the editors and the reviewers. Any product that may be evaluated in this article, or claim that may be made by its manufacturer, is not guaranteed or endorsed by the publisher.
