# Supplementary material for: Alterations in Emotional Diversity Correspond With Increased Severity of Attenuated Positive and Negative Symptoms in the Clinical High-Risk Syndrome
Source: Front Psychiatry. 2021 Dec 23;12:755027. doi: 10.3389/fpsyt.2021.755027 (PMC8732994; doi:10.3389/fpsyt.2021.755027)
Supplement: Supplementary file 1 [file Table_1.DOCX]

**Alterations in emotional diversity correspond with increased severity of attenuated positive and negative symptoms in the clinical high-risk syndrome**

**Zachary Anderson^1^, Tina Gupta^1^, William Revelle^1^, Claudia M. Haase^2^, Vijay A. Mittal^1^**

^1^Department of Psychology, Northwestern University

^2^School of Education and Social Policy, Northwestern University

*Corresponding Author:

Zachary Anderson

Department of Psychology

Northwestern University

Swift Hall 102, 2029 Sheridan Road

Evanston, IL 60208-2710

zacharyanderson2024@u.northwestern.edu

ORCID ID: 0000-0001-7253-8668

**Keywords:** Psychosis; clinical high-risk; attenuated positive symptom syndrome; emotion; emotional diversity

**S1**. An example calculation of emotional diversity with simulated data. This example is intended to illustrate fringe cases and their corresponding degrees of emotional diversity. This is also intended to provide code for the replication of this method.

## 1. Generate dummy cases

dummy1 <- c(1,1,1,1,1,1,1,1,1,1,1,1,1,1,1,1,1,1,1,1)
dummy2 <- c(4,4,4,4,4,4,4,4,4,4,4,4,4,4,4,4,4,4,4,4)
dummy3 <- c(4,1,1,1,1,1,1,1,1,1,1,1,1,1,1,1,1,1,1,1)
dummy4 <- c(1,4,4,4,4,4,4,4,4,4,4,4,4,4,4,4,4,4,4,4)
dummy5 <- sample(0:4,20,set.seed(42),replace = TRUE)

final_dummy <- data.frame(rbind(t(dummy1),t(dummy2),t(dummy3),t(dummy4),t(dummy5)))
colnames(final_dummy) <- c("amused","angry","ashamed","awe","scared","content","disgust","embarrassed","glad","grateful","hopeful","interested","love","proud","guilty","sad","contempt","sexual","surprise","sympathy")
final_dummy

## amused angry ashamed awe scared content disgust embarrassed glad grateful
## 1 1 1 1 1 1 1 1 1 1 1
## 2 4 4 4 4 4 4 4 4 4 4
## 3 4 1 1 1 1 1 1 1 1 1
## 4 1 4 4 4 4 4 4 4 4 4
## 5 0 4 0 0 1 3 1 1 0 3
## hopeful interested love proud guilty sad contempt sexual surprise sympathy
## 1 1 1 1 1 1 1 1 1 1 1
## 2 4 4 4 4 4 4 4 4 4 4
## 3 1 1 1 1 1 1 1 1 1 1
## 4 4 4 4 4 4 4 4 4 4 4
## 5 0 4 3 1 1 2 0 0 2 3

## 2. Logistic transform rescales data to a 0 to 1 scale

log_final_dummy <- logistic(final_dummy)
log_final_dummy

## amused angry ashamed awe scared content disgust
## 1 0.7310586 0.7310586 0.7310586 0.7310586 0.7310586 0.7310586 0.7310586
## 2 0.9820138 0.9820138 0.9820138 0.9820138 0.9820138 0.9820138 0.9820138
## 3 0.9820138 0.7310586 0.7310586 0.7310586 0.7310586 0.7310586 0.7310586
## 4 0.7310586 0.9820138 0.9820138 0.9820138 0.9820138 0.9820138 0.9820138
## 5 0.5000000 0.9820138 0.5000000 0.5000000 0.7310586 0.9525741 0.7310586
## embarrassed glad grateful hopeful interested love proud
## 1 0.7310586 0.7310586 0.7310586 0.7310586 0.7310586 0.7310586 0.7310586
## 2 0.9820138 0.9820138 0.9820138 0.9820138 0.9820138 0.9820138 0.9820138
## 3 0.7310586 0.7310586 0.7310586 0.7310586 0.7310586 0.7310586 0.7310586
## 4 0.9820138 0.9820138 0.9820138 0.9820138 0.9820138 0.9820138 0.9820138
## 5 0.7310586 0.5000000 0.9525741 0.5000000 0.9820138 0.9525741 0.7310586
## guilty sad contempt sexual surprise sympathy
## 1 0.7310586 0.7310586 0.7310586 0.7310586 0.7310586 0.7310586
## 2 0.9820138 0.9820138 0.9820138 0.9820138 0.9820138 0.9820138
## 3 0.7310586 0.7310586 0.7310586 0.7310586 0.7310586 0.7310586
## 4 0.9820138 0.9820138 0.9820138 0.9820138 0.9820138 0.9820138
## 5 0.7310586 0.8807971 0.5000000 0.5000000 0.8807971 0.9525741

## 3. Calculate relative proportions

row.total <- rowSums(log_final_dummy)
p <- log_final_dummy / row.total
p

## amused angry ashamed awe scared content disgust
## 1 0.05000000 0.05000000 0.05000000 0.05000000 0.05000000 0.05000000 0.05000000
## 2 0.05000000 0.05000000 0.05000000 0.05000000 0.05000000 0.05000000 0.05000000
## 3 0.06603049 0.04915629 0.04915629 0.04915629 0.04915629 0.04915629 0.04915629
## 4 0.03770419 0.05064715 0.05064715 0.05064715 0.05064715 0.05064715 0.05064715
## 5 0.03403395 0.06684362 0.03403395 0.03403395 0.04976163 0.06483973 0.04976163
## embarrassed glad grateful hopeful interested love proud
## 1 0.05000000 0.05000000 0.05000000 0.05000000 0.05000000 0.05000000 0.05000000
## 2 0.05000000 0.05000000 0.05000000 0.05000000 0.05000000 0.05000000 0.05000000
## 3 0.04915629 0.04915629 0.04915629 0.04915629 0.04915629 0.04915629 0.04915629
## 4 0.05064715 0.05064715 0.05064715 0.05064715 0.05064715 0.05064715 0.05064715
## 5 0.04976163 0.03403395 0.06483973 0.03403395 0.06684362 0.06483973 0.04976163
## guilty sad contempt sexual surprise sympathy
## 1 0.05000000 0.05000000 0.05000000 0.05000000 0.05000000 0.05000000
## 2 0.05000000 0.05000000 0.05000000 0.05000000 0.05000000 0.05000000
## 3 0.04915629 0.04915629 0.04915629 0.04915629 0.04915629 0.04915629
## 4 0.05064715 0.05064715 0.05064715 0.05064715 0.05064715 0.05064715
## 5 0.04976163 0.05995401 0.03403395 0.03403395 0.05995401 0.06483973

## 4. Calculate entropy (total emotional diversity)

entropy <- -rowSums(p * log(p))
entropy

## [1] 2.995732 2.995732 2.993264 2.993999 2.960573

### 4a Standardize entropy scores

entropy <- (entropy - mean(entropy)) / sd(entropy)
entropy

## [1] 0.5147734 0.5147734 0.3533736 0.4014510 -1.7843713

## 5. Calculate entropy (positive and negative emotional diversity)

p_positive = data.frame(cbind(p[,1],p[,4],p[,6],p[,9:14],p[,19]))
p_negative = data.frame(cbind(p[,2:3],p[,5],p[,7:8],p[,16:17]))

entropy_pos <- -rowSums(p_positive * log(p_positive))
entropy_neg <- -rowSums(p_negative * log(p_negative))

### 5a. Standardize entropy scores

entropy_pos <- (entropy_pos - mean(entropy_pos)) / sd(entropy_pos)
entropy_pos

## [1] 0.1271955 0.1271955 1.4788322 -1.2398853 -0.4933380

entropy_neg <- (entropy_neg - mean(entropy_neg)) / sd(entropy_neg)
entropy_neg

## [1] 0.4064028 0.4064028 -0.6065878 1.1776061 -1.3838239

**S2**. Correlations are presented between discrete emotion items across all attenuated positive and negative psychotic symptoms. Each correlation represents the mean zero-order correlation generated across a 10-fold cross validation procedure along with each estimates standard deviation.

| Unusual thought content and delusional ideas | Emotion Item | Mean correlation | Standard deviation of correlation |
| --- | --- | --- | --- |
|  | sad | 0.51 | 0.03 |
|  | angry | 0.5 | 0.03 |
|  | glad | -0.48 | 0.03 |
|  | guilty | 0.47 | 0.03 |
|  | scared | 0.47 | 0.02 |
|  | contempt | 0.44 | 0.03 |
|  | disgust | 0.42 | 0.04 |
|  | hopeful | -0.4 | 0.03 |
|  | embarrassed | 0.4 | 0.02 |
| Suspiciousness/persecutory ideas | guilty | 0.29 | 0.07 |
|  | scared | 0.26 | 0.03 |
|  | sad | 0.24 | 0.05 |
|  | embarrassed | 0.24 | 0.05 |
|  | proud | -0.17 | 0.03 |
|  | sexual | -0.15 | 0.08 |
| Grandiose ideas | glad | -0.4 | 0.03 |
|  | angry | 0.38 | 0.04 |
|  | sad | 0.37 | 0.05 |
|  | disgust | 0.35 | 0.05 |
|  | grateful | -0.34 | 0.04 |
|  | ashamed | 0.32 | 0.03 |
|  | content | -0.32 | 0.03 |
|  | guilty | 0.31 | 0.05 |
| Perceptual abnormalities and hallucinations | glad | -0.48 | 0.03 |
|  | angry | 0.46 | 0.03 |
|  | sad | 0.45 | 0.04 |
|  | disgust | 0.42 | 0.05 |
|  | guilty | 0.41 | 0.05 |
|  | hopeful | -0.41 | 0.03 |
|  | scared | 0.41 | 0.05 |
|  | ashamed | 0.4 | 0.04 |
| Disorganized communication | angry | 0.43 | 0.03 |
|  | scared | 0.4 | 0.04 |
|  | sad | 0.38 | 0.02 |
|  | glad | -0.38 | 0.04 |
|  | content | -0.37 | 0.03 |
|  | embarrassed | 0.35 | 0.02 |
|  | guilty | 0.35 | 0.02 |
|  | contempt | 0.34 | 0.03 |
| Social anhedonia | sad | 0.56 | 0.03 |
|  | angry | 0.47 | 0.02 |
|  | contempt | 0.45 | 0.03 |
|  | guilty | 0.45 | 0.03 |
|  | glad | -0.4 | 0.02 |
|  | disgust | 0.38 | 0.03 |
|  | amused | -0.38 | 0.03 |
|  | ashamed | 0.37 | 0.02 |
| Avolition | sad | 0.61 | 0.02 |
|  | angry | 0.57 | 0.02 |
|  | glad | -0.55 | 0.02 |
|  | scared | 0.5 | 0.04 |
|  | embarrassed | 0.45 | 0.05 |
|  | contempt | 0.43 | 0.04 |
|  | guilty | 0.42 | 0.04 |
| Expression of emotion | sad | 0.52 | 0.02 |
|  | angry | 0.47 | 0.03 |
|  | glad | -0.47 | 0.03 |
|  | contempt | 0.46 | 0.03 |
|  | disgust | 0.46 | 0.03 |
|  | grateful | -0.46 | 0.03 |
|  | scared | 0.44 | 0.03 |
|  | guilty | 0.44 | 0.02 |
| Experience of emotions and self | disgust | 0.59 | 0.02 |
|  | angry | 0.58 | 0.02 |
|  | sad | 0.57 | 0.02 |
|  | glad | -0.57 | 0.03 |
|  | ashamed | 0.55 | 0.03 |
|  | contempt | 0.53 | 0.04 |
|  | scared | 0.52 | 0.05 |
|  | guilty | 0.49 | 0.03 |
| Ideational richness | sad | 0.43 | 0.02 |
|  | angry | 0.41 | 0.03 |
|  | ashamed | 0.39 | 0.02 |
|  | contempt | 0.36 | 0.02 |
|  | guilty | 0.35 | 0.02 |
|  | glad | -0.33 | 0.04 |
|  | grateful | -0.32 | 0.04 |
|  | scared | 0.31 | 0.03 |
|  | disgust | 0.31 | 0.04 |
| Occupational functioning | sad | 0.56 | 0.02 |
|  | glad | -0.51 | 0.02 |
|  | angry | 0.5 | 0.02 |
|  | scared | 0.48 | 0.04 |
|  | contempt | 0.48 | 0.03 |
|  | guilty | 0.47 | 0.03 |
|  | ashamed | 0.43 | 0.04 |
|  | proud | -0.41 | 0.03 |
